# In-Network Processing of Joins in Wireless Sensor Networks

**DOI:** 10.3390/s130303358

**Published:** 2013-03-11

**Authors:** Hyunchul Kang

**Affiliations:** School of Computer Science and Engineering, Chung-Ang University, Seoul 156-756, Korea; E-Mail: hckang@cau.ac.kr

**Keywords:** join query, wireless sensor network, query processing, sensor network database

## Abstract

The join or correlated filtering of sensor readings is one of the fundamental query operations in wireless sensor networks (WSNs). Although the join in centralized or distributed databases is a well-researched problem, join processing in WSNs has quite different characteristics and is much more difficult to perform due to the lack of statistics on sensor readings and the resource constraints of sensor nodes. Since data transmission is orders of magnitude more costly than processing at a sensor node, in-network processing of joins is essential. In this paper, the state-of-the-art techniques for join implementation in WSNs are surveyed. The requirements and challenges, join types, and components of join implementation are described. The open issues for further research are identified.

## 1. Introduction

In wireless sensor networks (WSNs), sensor readings can be modeled as tuples of a *logical relation* which is physically distributed across the sensor nodes, and the retrieval of sensor readings can be expressed in relational queries. A *join* query that matches the tuples from two or more relations when they satisfy the given predicate conditions is one of major operations in relational databases. So is the join in WSN applications, which include vehicle surveillance and tracking, animal habitat monitoring, environment monitoring, home and commercial building automation, precision agriculture, and water resource management, to name just a few.

**Figure 1.**
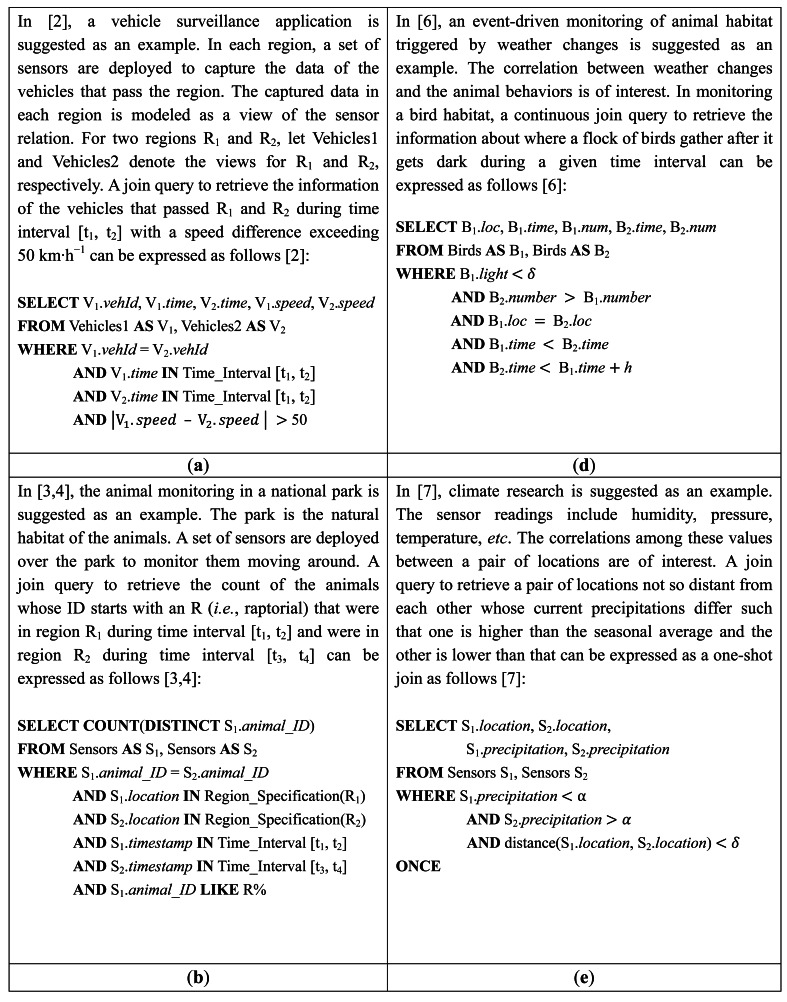

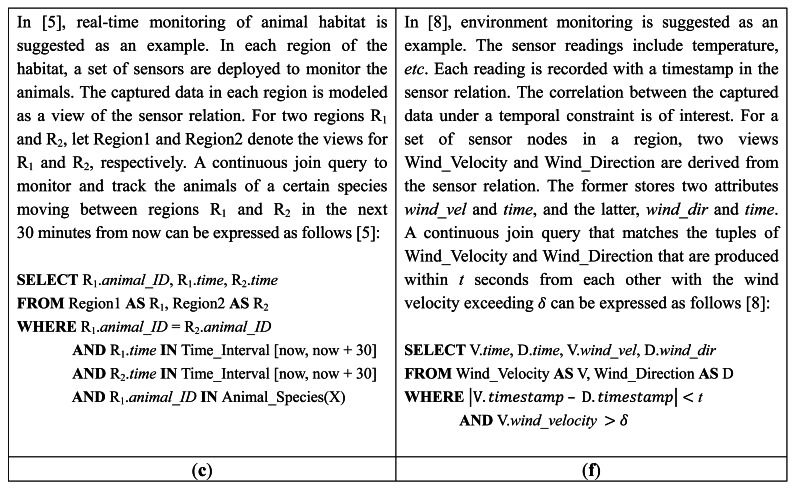
More examples of joins in WSN applications suggested in the literature. The examples of join queries in the literature are *not* shown. Refer to the referenced papers for them. Additional examples based on them in the same applications are given.

An example scenario of a join query in a river ecosystem monitoring application for water resource management, for instance, is as follows: the water quality management department is concerned about the pollution of a river, monitoring the pH values measured throughout the river. After detecting that the pH value in a region of the river has decreased below a certain threshold, which means that the acidity of the water is higher and the department needs to be alerted, the department is interested in checking whether such a phenomenon is now locally confined to the region or is possibly about to occur in the neighboring regions as well. The department would issue a query, “retrieve pH value and location for the neighboring regions of the river where the pH value decrease is detected.” This query can be declaratively expressed as a join, and given in SQL-like syntax as:

$$\begin{array}{l} \text{SELECT}\;{\text{S}}_{1}.pH,{\text{S}}_{1}.\text{location},{\text{S}}_{2}.pH,{\text{S}}_{2}.\text{location}\\ \text{FROM}\;\text{Sensors}\;\text{AS}\;{\text{S}}_{1},\text{Sensors}\;\text{AS}\;{\text{S}}_{2}\\ \text{WHERE}\;{\text{S}}_{1}.pH<\tau \;\text{AND}\;\text{distance}({\text{S}}_{1}.\text{location},{\text{S}}_{2}.\text{location})<\delta \end{array}$$

where Sensors in the FROM clause is the logical relation that comprises the river data tuples sampled by the sensors deployed along the river, and the condition with the function distance() in the WHERE clause is the join predicate. More examples of joins in WSN applications suggested in the literature are given in Figure 1.

In WSNs, data transmission is orders of magnitude more costly than processing on a sensor node [1]. Given a join query, therefore, *in-network* processing where join operators are pushed into the network such that the amount of data transmission could be considerably reduced is essential. Although the processing of joins in the conventional centralized or distributed databases is a well-known problem, that in WSNs has quite different requirements and challenges. First, the lack of statistics on sensor readings makes query optimization very difficult. Second, a naïve implementation could expend much energy in transmitting data, severely shortening the network lifetime. Third, limited resources (computing power and memory) in sensor nodes make ordinary algorithmic approaches infeasible. Fourth, node or link failures could greatly undermine the correctness of the join result.

In this paper, we present a survey of the state-of-the-art techniques of in-network join processing in WSNs. This topic is a young one, receiving much attention in recent years. We think that a meaningful amount of work has been accumulated in the literature, and that now it is timely to collate the various techniques for classification and comparison. To the best of our knowledge, our survey is the first one on this topic.

The rest of this paper is organized as follows: in Section 2, we present the background materials for this paper: sensor networks, their modeling as a relational database, declarative query expression, external join, in-network query processing, join types and implementations in the conventional databases, and sliding window join. In Section 3, we give an overview of join processing in WSNs: requirements and challenges, join types, and components of join implementation. In Section 4, we describe the state-of-the-art techniques of in-network join processing proposed in the literature. In Section 5, we discuss the open issues with future research directions. Finally, we conclude in Section 6.

## 2. Preliminaries

In this section, we present the background materials on a sensor network, its modeling as a relational database, declarative query expression, external join, in-network join processing, join types and implementations in the conventional databases, and sliding window join.

### 2.1. Sensor Network and Database

We consider a sensor network that consists of a set of sensor nodes. Each node is a battery-powered small computer with limited processing capability and memory capacity. It is equipped with one or more sensing devices and a radio for wireless communication. A node can directly communicate with other nodes within its wireless range. For multi-hop communication with other nodes beyond the wireless range of a node, the nodes form a network. The power in a node is also a limited resource. The power consumption in a node is dominated by data transmission. It is orders of magnitude more costly than processing on a node [1]. Power conservation (or energy-efficiency) is one of the most important requirements when a sensor network performs a task. The longevity of a sensor network depends on it. A sensor network is often ad-hoc and unstable, and each node has limited knowledge of other nodes and how they are connected. Finally, we assume that a sensor network is connected to the *base station* which is not resource constrained.

In WSNs, the data acquired by a sensor node at an instant in time is the readings of one or more sensors on the node (e.g., temperature, humidity, *etc*.) These readings can be modeled as a relational tuple that has one attribute per sensor. Each attribute is called a *sensor attribute* and the resulting tuple is called a *sensor tuple*. Besides sensor attributes, a sensor tuple is often modeled as having *auxiliary attributes* such as node ID, geographic coordinates of the node, and the timestamp of the sensor readings. The *stream* of sensor tuples in WSNs can be modeled as a relation. It is called a *sensor relation*. Logically, it has at most one sensor tuple per node per instant in time. Physically, it is horizontally partitioned across the sensor nodes in WSNs. A composite of the node ID and timestamp attributes could be designated as a primary key of a sensor relation. The sensor nodes could be heterogeneous, having different sets of sensing devices. In a sensor tuple produced in a node without a particular sensing device, the corresponding sensor attribute is set to NULL. Depending on the degree of heterogeneity, different groups of sensor nodes could be modeled as separate sensor relations, one per group. Views of a sensor relation can also be defined as in relational databases.

A query joining sensor readings can be declaratively expressed against the sensor relations in SQL-like syntax as shown in the previous section and in Figure 1. The sensor relations and/or its views are listed in the FROM clause as join operand relations. For example, in the query expressions in Figure 1(b,d,e), the sensor relations are directly queried. In the query expressions in Figure 1(a,c,f), views of the sensor relation are queried. The join predicates of interest are specified in the WHERE clause often with selection predicates. Each predicate is either on the sensor attributes or on the auxiliary attributes. In the query expression in Figure 1(d), for example, there are five predicate conditions in the WHERE clause: The first one (B_1_.*light* < *δ*) is a selection predicate on a sensor attribute *light*. The second one (B_2_.*num* > B_1_.*num*) is a join predicate on a sensor attribute *num* (*i.e.*, number). The remaining three (B_1_.*loc* = B_2_.*loc*; B_1_.*time* < B_2_.*time*; B_2_.*time* < B_1_.*time* + *h*) are join predicates on auxiliary attributes *loc* (*i.e.*, geographic location of a sensor node) and *time* (*i.e.*, timestamp of a sensor tuple). The predicates on sensor attributes are the ordinary ones used in the conventional database queries. The predicates on auxiliary attributes add spatial as well as temporal characteristics to the join queries in WSNs.

Since a join is expressed declaratively, its implementation strategy needs to be devised. A straightforward implementation of a join query in WSNs is to perform the *external join*: Every sensor node that participates in the query transmits all the sensor tuples satisfying the selection predicates to the base station where the join is computed. Since the external join transmits non-joinable tuples as well, it would not be energy-efficient when the join is highly selective, *i.e.*, join selectivity is very low (for a join *R* ⨝ *S*, join selectivity (or join selectivity factor) is defined to be $\frac{\left|R⨝S\right|}{\left|R\right|\cdot \left|S\right|}$ [9].

It would incur much more communication than necessary, depleting the battery of sensor nodes severely. In-network join processing could be a more efficient implementation. In in-network query processing, query operators are pushed into the network. Their executions are distributed across the sensor nodes of the network, consuming the sensor tuples and producing intermediate query results. The sensor tuples are filtered, aggregated, or cross-referenced inside the network instead of being transmitted to the base station. With in-network join processing, the amount of data transmission could be considerably reduced compared to the external join.

### 2.2. Join Types and Implementations in Conventional Databases

In a relational database, join queries can be expressed on various types of join predicates. The most widely used join type is a *theta-join*, *R* ⨝*_AθB_ S*, in which the join predicate is *R. A θ S. B* where *θ* is one of the following comparison operators: <, >, ≤, ≥, =, and ≠. When *θ* is =, it is called an *equi-join*.

The three major implementations for these join types in the conventional databases are *nested-loop join*, *hash join*, and *sort-merge join* [10]. In this section, we briefly review each implementation. They are adapted to in-network processing of joins in WSNs [2–5,8,11,12] as described in Section 4.

#### Nested-Loop Join

Given a theta-join *R* ⨝*_AθB_ S*, it matches *R* and *S* tuples that satisfy the join predicate out of the Cartesian product of *R* and *S* through nested loops. In the *outer* loop, the tuples of *R* are scanned. For each tuple of *R*, the tuples of *S* are scanned in the *inner* loop. *R* is called an *outer relation* and *S* an *inner relation*. If there is an index on *S.A*, the tuple matching in the inner loop can use it. Such an implementation is called an *indexed nested-loop join*.

#### Hash Join

Given an equi-join *R* ⨝*_A=B_ S*, it first scans *R* (*S*) and partitions it to *k* buckets *R*_1_, …, *R_k_* (*S*_1_,…, *S_k_*) using a hash function on the joining attribute *A* (*B*). Then, it carries out the matching of *R* and *S* tuples that satisfy the join predicate only between bucket pairs *R_i_* and *S_i_*, *i* = 1, …, *k*.

#### Sort-Merge Join

Given an equi-join query *R* ⨝*_A=B_ S*, it first sorts the tuples of *R* (*S*) on the joining attribute *A* (*B*). Then, it carries out the matching of *R* and *S* tuples that satisfy the join predicate by scanning *R* and *S* in one pass each.

### 2.3. Semijoin in Distributed Databases

In distributed databases, a join *R* ⨝*_A=B_ S* could be a distributed one: *R* and *S* reside at different sites, and the join result is to be consumed at a third site (*i.e.*, result site). There could be several implementations of this join, each of which would incur different amounts of data transmission. In a naïve one, *R* and *S* are respectively sent to the result site, where the final join is conducted. The external join in WSNs corresponds to this naïve implementation. In another one, *semijoins* [13] are executed first to filter out non-joinable tuples of *R* and *S*. Then, only those tuples of *R* and *S* that have survived (*i.e.*, joinable) are sent to the result site for the final join.

Given a distributed join *R* ⨝*_A=A_ S*, two semijoins are possible: *R* ⋊*_A=A_ S* and *R* ⋉*_A=A_ S*. The semijoin *R* ⋊*_A=A_ S* is executed in three steps: (1) At the site of *R*, the set of values of the joining attribute *A* of *R* (*i.e.*, *R*[*A*]) is retrieved; (2) *R*[*A*] is sent to the site of *S*; (3) At the site of *S*, *R*[*A*] is joined with *S* to produce *R* ⋊*_A=A_ S*.

*R* ⋊*_A=A_ S* is a subset of *S*. It contains only those tuples of *S* that are joinable with *R* on *A*. A semijoin could be employed in processing a distributed join if it is *cost-effective* in communication [13,14]. For *R* ⋊*_A=A_ S*, its cost is defined to be the cost of sending *R*[*A*] while its benefit is defined to be the difference between the cost of sending S and that of sending *R* ⋊*_A=A_ S*. A semijoin is said to be *cost-effective* if the benefit exceeds the cost. To figure out if a semijoin is cost-effective, the *semijoin selectivity* (or *semijoin selectivity factor*) of a relation on the joining attribute needs to be estimated. Let *f_R_A__* denote the semijoin selectivity of relation *R* on joining attribute *A*. *f_R_A__* is the probability that a value in *A* appears in *R* assuming that the values of *A* are uniformly and independently distributed on the join operand relations [14]. It is estimated as ||*R[A]*|/|*D_A_*|, where *D_A_* is the domain of *A* [15]. Given a join *R* ⨝*_A=A_ S*, *f_R_A__* indicates what fraction of *S* would join with *R* on *A* (*i.e.*,| *R* ⋊*_A=A_ S* | = *f_R_A__* · |*S*|).

Given a distributed join query *q* = *R* ⨝*_A=A_ S* ⨝*_B=B_T*, a semijoin, *R* ⋊*_A=A_ S*, produces *S’* It is obtained after eliminating all the tuples of *S* not joinable with *R* on *A*. This semijoin is said to *reduce S* to *S*’. Since *S’* might contain those tuples not joinable with *T* on *B*, *S*’is a *partial reduction* of *S*. A subsequent semijoin, *T* ⋊_B_ *_=B_S’* produces *S’*. Since it contains only those tuples necessary for the result of *q* without any superfluous non-joinable tuples, *S’* is the *full reduction* of *S*.

### 2.4. Sliding Window Join in Data Stream Management Systems

A full evaluation of a join between two unbounded streams of tuples is not practical because unbounded memory is required. In data stream management systems, a join of two streams *R* and *S* are often modeled as a *sliding window join* [16–18]. In a sliding window join of *R* and *S*, a newly arriving tuple of *R* could produce join results only with the tuples of *S* that belong to a window of *S*. The window could be either *time-based* or *tuple-based* [19]. A time-based sliding window join of *R* and *S* works as follows. For a tuple *r* of *R* arriving at time instant *t*, let w*_S_* (*t*, *h_S_*) denote the window of *S* for *r* where *h_S_* is the window size. w*_S_*(*t*, *h_S_*) is defined to contain those tuples of *S* that have arrived in the time interval [*t−h_S_*, *t*]. A sliding window of *R* is defined similarly, and a newly arriving tuple of *S* is joined only with the sliding window of *R*. In a tuple-based sliding window join, the window of *R* (*S*) is defined to contain the last *n* tuples of *R* (*S*), where *n* is a positive integer associated with the window.

## 3. Overview of Join Processing in WSNs

The problem of join processing in WSNs is quite different from that in the conventional centralized or distributed databases. In this section, we address the requirements and challenges of in-network join processing in WSNs, present join types characterized by spatial as well as temporal predicates, and describe components of join implementation.

### 3.1. Requirements and Challenges

Due to the constraints of WSNs stated in Section 2.1, join implementation in WSNs is very complicated, whose optimization is very difficult. Major requirements and the resulting challenges are described below.

#### Stream processing

In the conventional databases, join operand relations are stored ones. Access paths to and the statistics on them including the statistics on the joining attributes are available. In WSNs, join operand relations are streams of sensor readings. The challenges are to execute a stream join using limited resources of sensor nodes, and to collect the statistics on the sensor readings for cost-based query optimization.

#### Distributed processing

Because of the memory constraints in the sensor nodes, it would not be possible to collect all the tuples at one designated node in the network for join processing. Thus, distributed processing is required. As stated in Section 2.1, in WSNs, each node has limited knowledge of other nodes and the network connectivity. Therefore, the problem of generating an efficient distributed strategy is a difficult challenge.

#### Protocol-oriented implementation

Since the sensor nodes in WSNs are only with constrained resources, a join implementation which is free from any physical error that might be caused due to the resource constraints while guaranteeing algorithmic correctness of the join result is required. In transmitting data among the sensor nodes, for example, an overflow at a receiving node due to its memory constraints should be avoided. A carefully designed protocol by which data transmission as well as query operations are synchronized is required. Such a protocol-oriented implementation under the resource constraints is a crucial challenge.

#### Fault-tolerance

As stated in Section 2.1, WSNs are often unstable. Node or link failures can occur or several reasons. Even if just a small number of joinable tuples are missing because of a failure, it could have a great effect on the join result. Dealing with failures is a challenge because fault tolerance with duplicate transmissions, retransmissions, and exchanges of control information that can help detect failures would not only increase the complexity of join processing but requires more communication.

### 3.2. Join Types

A join query in WSNs is often characterized by its selection/join predicates on the *auxiliary* attributes. The auxiliary attributes such as the location of a sensor node and the timestamp of the sensor readings represent the spatio-temporal dimension of the sensor readings. The spatio-temporal predicates on these attributes are important factors of a join query that affect its implementation.

#### Spatial predicates

For a join query *R* ⨝ *S*, let ℝ ( 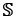) denote the set of sensor nodes which produce the tuple stream of *R* (*S*). Table 1 summarizes notations in the paper. A spatial predicate in a join query is the one on the attribute *location* (*i.e.*, geographic coordinates) of the sensor nodes. It restricts the sensor nodes that are to participate in the query. Spatial predicates can be specified as selection predicates or as join predicates. An example of the former is the predicate “S_1_.*location* **IN** Region_Specification(R_1_)” in the WHERE clause of the query in Figure 1(b). An example of the latter is the predicate “distance(S_1_.location, S_2_.location) < *δ*” in the WHERE clause of the query in Figure 1(e).

**Figure 2.**
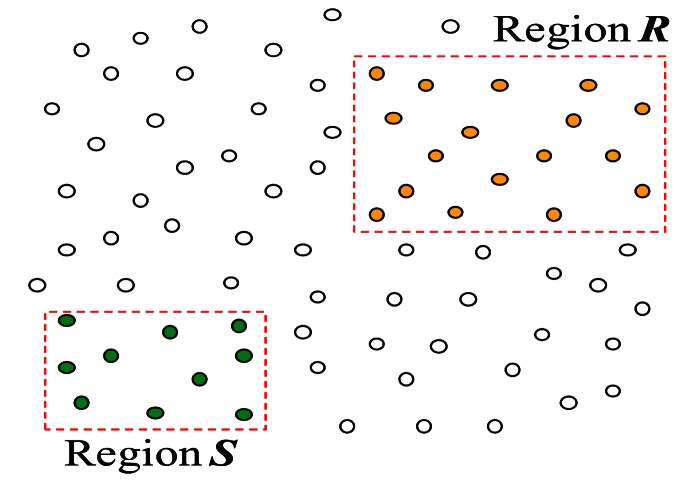
Inter-region join *R* ⨝ *S*, where each small circle denotes a sensor node.

For a join query *R* ⨝ *S*, only selection spatial predicates on *R* (and/or on *S*) can be specified. Queries in Figure 1(a–c) are of such type. Since there is no join spatial predicate, ℝ and 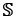 can be easily identified assuming that each node knows its location. Since ℝ ( 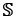) covers a fixed *region* of the network denoted as ***R*** (***S***), such type of join is an *inter-region join*. An instance of an inter-region join is shown in Figure 2. In an inter-region join *R* ⨝ *S*, it is typical that regions ***R*** and ***S*** do not overlap with each other as shown in Figure 2. An important property of an inter-region join is that a node that has received an inter-region join query can determine if it is to participate in the query without communicating with other nodes. The processing of an inter-region join has received so far the most attention in the literature [2–5,8,11,12,20,21], and several techniques that are suitable for such a join type have been proposed.

If a join query specifies *join* spatial predicates, they not just restrict the sensor nodes that are to participate in the query but also restrict *pairing* among the sensor nodes for the join. Queries in Figure 1(d,e) are of such type. In general, a node that has received this type of query cannot determine if it is to participate in the query without communicating with other nodes. This could make in-network join implementation more difficult compared to an inter-region join. This type of join query was investigated in [6,7,22–24].

#### Temporal predicates

Since a sensor relation is an *unbounded* stream of sensor tuples, a full evaluation of a join between two sensor relations is neither possible nor practical. For a sensor relation *R*, let w*_R_* denote a *time-based* window [19] of *R* (in this section, we mention only the time-based window because we assume that temporal predicates are specified on the timestamp attribute. The description of the join types associated with temporal predicates in this section can be extended to incorporate the tuple-based window as well. w*_R_* is a subset of *R* restricted by a time interval. A join query *R* ⨝ *S* in WSNs is required to specify w*_R_* and w*_S_* so that each tuple of *R* (*S*) may be joined with w*_S_* (w*_R_*) instead of with *S* (*R*). Selection predicates or join predicates on the *timestamp* of the sensor readings can specify such a window as described below.

Three types of windows can be specified: *fixed*, *sliding*, and *jumping*. The time interval of a *fixed* window is specified by *selection* predicates on timestamps. For a join *R* ⨝ *S* to be executed as a *one-shot* query, a pair of fixed windows w̿*_R_* and w̿*_S_* are specified such that the join result is w̿*_R_* ⨝ w̿*_S_*. The time interval [*t_1_*, *t_2_*] associated with w̿*_R_* (or w̿*_S_*) is specified such that *t_1_*≤ *t_2_* ≤ *t*, where *t* is the point of query submission in time. The queries in Figure 1(a,b) is for such a one-shot join [2–4]. Note that w̿*_R_* and w̿*_S_* are *static* (*i.e.*, stored) rather than *streaming* in such a one-shot join.

Fixed windows can be used for a *continuous* join as well if their time intervals are the future ones. The example query in Figure 1(c) is for such a continuous join [5], where each window contains the tuples that are to be produced in the next 30 minutes. Thus, the windows in such a continuous join are streaming ones, and the window size (*i.e.*, 30 minutes) is the *lifetime* of the query.

The time interval of a *sliding* window is specified by *join* predicates on timestamps. For a join *R* ⨝ *S* to be executed as a continuous query, a pair of sliding windows w̃*_R_* and w̃*_S_* are specified such that the join is modeled as a *time-based sliding window join* [19]. This model of continuous join in WSNs has received much attention in the literature [6,8,11,20,22,23].

A special case of a sliding window join is when one of w̃*_R_* and w̃*_S_* is not specified. For a continuous join *R* ⨝ *S* where a tuple of *R* (e.g., representing an event *e_r_*) is joined only with the future tuples of *S* (e.g., representing future events *e_s_* related to *e_r_*) but not with the past tuples of *S*, only one sliding window w̃*_R_* needs to be specified. When a tuple of *S* is produced, it is joined with w̃*_R_*. However, it is not kept because no window of *S* is maintained. When a tuple of *R* is produced, it is not joined with *S* at the moment but kept in w̃*_R_*. The example query in Figure 1(d) is for such a continuous join [6].

For a join *R* ⨝ *S* modeled as a time-based sliding window join, the window w̃*_R_* (w̃*_S_*) is already a stored subset of *R* (*S*) when it is scanned for a join with a tuple of *S* (*R*) just produced. However, we regard w̃*_R_* and w̃*_S_* as streaming because they are sliding continuously.

In the context of *acquisitional* query processing in TinyDB [25], a one-shot join query can be expressed *without* temporal predicates. In the query in Figure 1(e), for example, no temporal predicate is specified in the WHERE clause. It is a one-shot join query *R* ⨝ *S* as declared by the ONCE clause [26]. Its result is *R_c_* ⨝ *S_c_*, where *R_c_* and *S_c_* respectively contain only the current measurements (*i.e.*, the most recently sampled tuples of *R* and *S*). In other words, *R_c_* and *S_c_* have one tuple per node in ℝ and 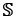, respectively. Therefore, *R_c_* and *S_c_* are static. This type of query was considered in [7].

A continuous join query can also be expressed without temporal predicates in TinyDB. Let us consider the following query:

$$\begin{array}{l} \text{SELECT}\;{\text{S}}_{1}.\text{location},{\text{S}}_{2}.\text{location},{\text{S}}_{1}.\text{precipitation},{\text{S}}_{2}.\text{precipitation}\\ \text{FROM}\;\text{Sensors}\;{\text{S}}_{1},\text{Sensors}\;{\text{S}}_{2}\\ \text{WHERE}\;{\text{S}}_{1}.\text{precipitation}<\alpha \;\text{AND}\;{\text{S}}_{2}.\text{precipitation}>\alpha \;\text{AND}\;{\text{distances}(\text{S}}_{1}.\text{location},{\text{S}}_{2}.\text{location})<\delta \\ \text{SAMPLE PERIOD}\;10\text{s} \end{array}$$

This query is the same as the one in Figure 1(e) except that the ONCE clause is replaced by the SAMPLE PERIOD clause [26]. This clause specifies a jumping window, defining the period of time between the start of each sample period such that tuples are produced at well-defined intervals. In the above query, the sample period is set to 10 seconds and the join is executed once every 10 seconds. In such a *periodic* continuous query, the join in each execution is treated as a one-shot query as if the ONCE clause were specified. Periodic continuous join queries including this type were investigated in [11,21,24].

### 3.3. Components of Join Implementation

An implementation of a join in WSNs consists of several components. Some of the major ones that are common to most of the techniques surveyed in this paper are described below.

#### Routing Protocol

**Figure 3.**
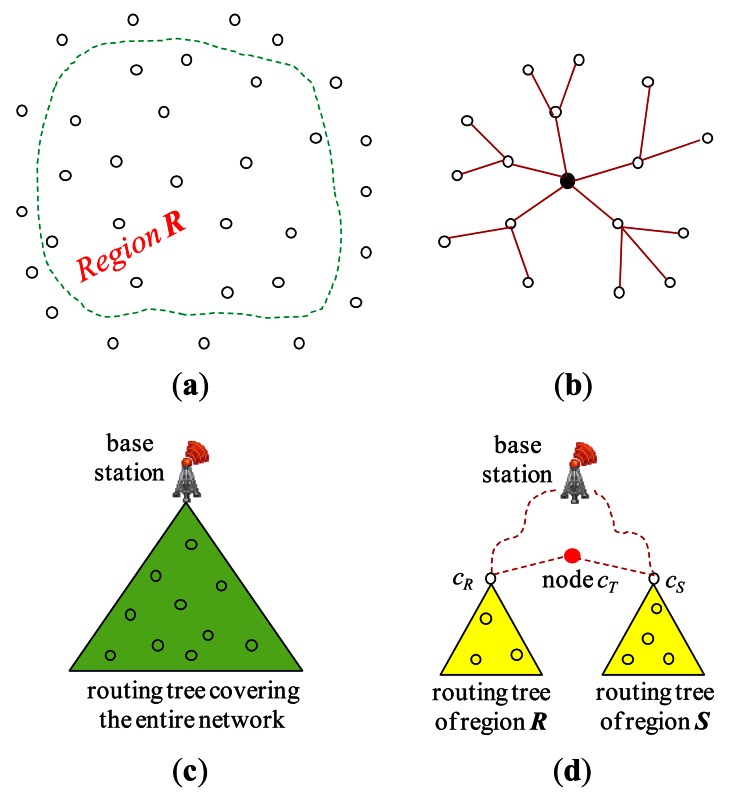
(**a**) A region, ***R***, of the sensor network that consists of sensor nodes, where each small circle denotes a sensor node; (**b**) A routing tree constructed for region ***R***, where the sensor node at the center (*i.e.*, a dark circle) is chosen as the root; (**c**) A routing tree constructed for all the sensor nodes of the network with the base station as the root; (**d**) Join initiation for an inter-region join *R* ⨝ *S*.

Throughout join processing from query dissemination to the delivery of the final join result, a routing protocol is required. Among others, the tuples and/or joining attribute values need to be shipped and/or replaced inside the network with a routing protocol to execute join operators. For this purpose, the standard *tree routing* protocol presented in [25] is widely employed. Figure 3(a) shows a set of sensor nodes in a region, ***R***, of the sensor network and Figure 3(b) an example of a routing tree constructed for them with the node at the center as the root. When all the nodes of the sensor network participates in the query, a routing tree covering all the sensor nodes is constructed with the base station as the root (Figure 3(c)). For an inter-region join *R* ⨝ *S*, a routing tree per region could be constructed. Figure 3(d) shows the two routing trees for regions ***R*** and ***S***. The location routing protocol such as GPSR [27] is also widely employed when routing data to the sensor node nearest to given geographic coordinates. It is usually used in combination with geographic hashing [28].

#### Query dissemination

The *query sink* where a join query is submitted is either the base station or a node inside the network. The query message is supposed to be delivered to every node of the network. For an inter-region join *R* ⨝ *S*, however, the query message can be routed to the *center* coordinates of region ***R*** (***S***) using a location routing protocol like GPSR. The query is received at the sensor node *c_R_* (*c_S_*) nearest to the center coordinates of region ***R*** (***S***). In region ***R*** (***S***), a routing tree whose root is *c_R_* (*c_S_*) is constructed (Figure 3(d)) using the standard method of [25] while the query is disseminated in the region.

#### Join initiation

After a node receives a join query, it might need to participate in the process of join initiation. For an inter-region join, first the relevant information could be gathered through the routing tree of each region, and then collected at a sensor node *c_T_* where a join processing strategy is dynamically generated. *c_T_* could be a midpoint node between the two roots [5,12] or a node in the designated region where the join is to be conducted [3,4]. The strategy is notified back to the two roots, and this completes join initiation. Figure 3(d) shows the node *c_T_* and the two routing trees for regions ***R*** and ***S***. The selection of the *join node* where the join is to be conducted for an inter-region join is also a function of join initiation. If the memory space of one node is not sufficient for the join, a set of nodes are selected. They form a *join region*. The location of a join region could be computed in the query sink [3,4,8,11].

Join initiation for other types of join queries was investigated in [22,23]. In the technique called *Pair-wise Join* that will be described in Section 4.2, *multiple* routing trees are employed for finding out the optimal path between a pair of nodes that participate in the join and the optimal join node on it.

#### Filtering

As described in Section 2.3, in the conventional distributed databases, the semijoin and its variants are employed to filter out non-joinable tuples before the final join is conducted. They reduce the amount of data to be transmitted [9,14]. The same principle could be exploited for joins in WSNs. Such techniques conduct in-network filtering of non-joinable attribute values and/or of non-joinable tuples using semijoins or its variants [2,4–7,12,21]. An alternative approach is a stochastic one using and maintaining a join filter in every sensor node [24]. This technique called *Continuous Join Filtering (CJF)* is described in Section 4.3.

Given a 2-way join *R* ⨝ *S* in WSNs, the techniques proposed in the literature fall into the following three categories based on the reduction of the join operand relations:

- Full reduction of both operand relations [2,4,21]
- Full reduction of one operand relation [6]
- Partial reduction of both operand relations [5,7,12,24]

#### Involvement of base station

In all the techniques surveyed, after non-joinable tuples are filtered out if such a step exists, those tuples that have survived are sent either to the base station or to the query sink inside the network or to the designated join region of the network for the final join. Excluding this final join phase, all the techniques except those of [6,7,24] conduct in-network processing of join *without* any form of involvement of the base station. In the techniques of [6,7,24], the base station is not just involved but plays a coordinating and core role.

#### Adapted join implementation

Most of the proposed techniques can be regarded as having some components that are variations or extensions of the join or semijoin implementations in the conventional centralized/distributed databases. They are adapted to the WSN environments. The techniques in [3,4,8,11] adapt nested-loop join or indexed nested-loop join. Those of [2,5,8] adapt hash join, partitioning relations into buckets which are replaced inside the network. Even the sort-merge join is logically adapted in the techniques of [5,12] though no physical sorting is carried out in the network. The techniques of [2,4–7,12,21] conduct in-network filtering of non-joinable tuples with the semijoin or its variations adapted to WSNs.

#### Cost-based optimization

Assuming that the statistics on the sensor readings are given a priori or collected for a long-running continuous join, cost-based join optimization is carried out. The list includes:

- computation of the optimal join region for an inter-region join [4,11,20]
- selection of the optimal inner/outer relation in an adapted nested-loop join implementation [3]
- selection of the optimal join path in the network and the optimal join node on the path for a pair of nodes that participate in a join [22,23]
- adaptive re-optimization for the join path and the join node for a pair of nodes that participate in a join [22]
- adaptive re-optimization for multiple pairs of nodes that participate in a join [22]
- selection of the optimal in-network semijoin strategy for a routing tree based inter-region join [21]
- maintenance of the optimal join filters [24]

All of the aforementioned cost-based optimizations except that in [3] are suitable for a long-running continuous join query. One reason is that they require the statistics on the sensor readings for cost analysis and their meaningful values could not be collected soon. Another reason is that the cost incurred for such an optimization might be so high that it needs to be amortized during the lifetime of the query.

#### Collection of statistics

To conduct cost-based optimizations, the statistics on sensor relations and join result are required. They include the size of join operand relations, the size of join result (or join selectivity), semijoin selectivities, and selectivity of each selection predicate on sensor attributes, and so on. The collection of the statistics is a part of join implementation. In [11], for a periodic continuous join, the initial execution is assumed to be carried out at the query sink to sample the statistics. In [21], the external join is employed in the learning phases to collect the statistics. In [20,22,23], the statistics are assumed to be given through the executions of a long-running continuous join.

#### Failure handling

In the techniques employing tree routing, failures are handled by restructuring the tree [6,7,22,23]. For this, each node is supposed to maintain information on its neighbor nodes and to send a heartbeat message periodically to its neighbor nodes. The techniques employing the location routing protocol like GPSR [4,8,11] can adapt to node failures because such protocols can handle node failures, searching for new routes if a node on a route fails and guaranteeing packet delivery as long as a route exists. Data replication can also be employed to handle node failures [8].

#### Duration of join execution

It is a component of a join query and therefore relevant to its implementation. Although a continuous query is not necessarily a long-running one, it is often so in many applications (e.g., long-term monitoring). In implementing a long-running continuous join, the opportunities of collecting and maintaining the time-varying statistics for cost-based optimization [11,20–22] and of improving the strategy for the subsequent executions [6,24] should be exploited.

## 4. Techniques of In-Network Join Processing

In this section, we present the state-of-the-art techniques of in-network join processing in WSNs proposed in the literature. In doing so, we elaborate upon each technique by emphasizing its characteristics and salient features. In Section 4.1, we first briefly review some of the first approaches [20,25,29,30]. In Section 4.2, we present a group of techniques which do not consider the filtering of non-joinable tuples before the final join [3,4,8,11,22,23]. In Section 4.3, we present another group of techniques. One of their main features is the filtering of non-joinable tuples using a semijoin or its variants [2,4–7,12,21,24]. In Section 4.4, we summarize and assess the techniques surveyed in Sections 4.2 and 4.3. In particular, Table 2 in Section 4.4 compares the surveyed techniques where each row can be referred to as a summary of the corresponding technique.

### 4.1. First Approaches

In [29], Yao and Gehrke compared the communication cost of the external join with that of an in-network join implementation through an experiment. They showed that the performance could differ significantly in favor of as well as against the in-network join depending on the join selectivity. In the in-network join, it was assumed that the join is conducted in a single node of the network where all the tuples are collected. The optimal location of the join node was not addressed.

In [20], Bonfils and Bonnet investigated the problem of searching for the optimal join node assuming that the join is conducted in a single node. They considered an inter-region join *R* ⨝ *S* whose result is to be consumed at the query sink. The join is a long-running continuous one modeled as a window join. Assuming that the data rates (or the sizes) of *R*, *S*, and *R* ⨝ *S* are known and that the communication cost between two sensor nodes is known, *C_a_*(*N*), the join assignment cost of node *N* which is the cost of assigning the join operator to a node *N* is computed [20]. Initially, a node *N_0_* is randomly selected as the join node. Afterwards, a neighbor node *N*_1_ of *N*_0_ is checked. If *N*_1_ has lower assignment cost than *N*_0_ (*i.e.*, *C_a_*(*N*_1_) < *C_a_*(*N*_0_)), *N*_1_ becomes a new join node. This way, the location of the join node progressively moves towards its optimal location. Also, the optimal location of the join node would change adaptively as the data rates and selectivities change.

In [25], Madden *et al*. considered a join between a *storage point* and the sensor relation. A storage point corresponds to a materialized view in relational databases or to a materialization point in data stream systems. It buffers a stream of sensor tuples that belong to a window specified by a query. A storage point is materialized at a single node. The join is implemented by a nested-loop join where the sensor relation is the outer relation and the storage point is the inner one. The tuples of the sensor relation are transmitted through a routing tree.

In [30], Abadi *et al*. investigated a join between an *external relation* and the sensor relation. The former stores the tuples that specify the conditions of the events to detect in the network. This relation is pushed into the network for the join. There are three cases depending on the size of the external relation. First, it can be stored at a node. Second, it can be partitioned into a group of nodes that are within 1 hop distance (intermediate join). Third, it is too large to fit in the group of nodes in the second case (large join). For an intermediate join, the techniques of group formation, relation partition, and distributed join were proposed. For a large join, the techniques of non-joinable tuple filtering using a Bloom filter [31], partial join, and cache diffusion were proposed.

In these first approaches, the join was assumed to be performed in a single node [20,25,29] or one of the join operand relations was not a sensor relation [30]. In the next two subsections, the techniques of a join among the sensor readings that do not make the above assumption are presented.

### 4.2. Techniques without Filtering of Non-Joinable Tuples

In this section, we present a survey of the join implementations named *Distribute-Broadcast Join* [11], *Mediated Join* [3,4], *Distributed Index-Join* [8], *Distributed Hash-Join* [8], and *Pair-wise Join* [22,23]. They do not support non-joinable tuple filtering before the final join. They are focusing on other issues including adaptation of the conventional join implementations to WSNs, the optimal location of the join nodes, indexing, and the optimal join initiation.

#### Distribute-Broadcast Join

In [11], Chowdhary and Gupta considered an inter-region join *R* ⨝ *S* to be executed as a continuous join query. They considered two query models. First, *R* and *S* are assumed to be *static* and the execution of such a static join is repeated. Second, *R* and *S* are *streaming* and the join is modeled as a sliding window join.

**Figure 4.**
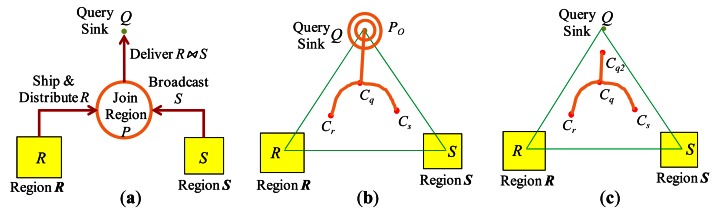
(**a**) Distribute-Broadcast Join; (**b**) A possible shape of an optimal join region; (**c**) Another possible shape of an optimal join region.

In the first model, join processing proceeds as follows: All the tuples of *R* are shipped and distributed uniformly in one or more nodes that are located in a designated region *P* of the network, which is called a *join region*. Then, the tuples of *S* are broadcast to *P*. At each node of *P*, join processing is performed and the matched tuple pairs are transmitted to the query sink. In such a protocol called *Distribute-Broadcast Join* (shown in Figure 4(a)), *R* and *S* respectively play the role of the inner and the outer relation of a nested-loop join.

In [11], the *optimal* join region with which the total communication cost for *Distribute-Broadcast Join* is minimized was investigated. If the join region is to be simply constructed as a *circular region* around a point *C* in the network, the optimal location of *C* is determined such that the weighted sum of the distances from *C* to region ***R***, to region ***S***, and to the query sink *Q* is minimized, where the weights are the sizes of *R*, *S*, and *R* ⨝ *S*. Such an optimal point *C* lies within the triangle formed by region ***R***, region ***S***, and the query sink *Q* (*Δ****RS****Q*), and this particular join implementation is called *Centroid Join*.

If the join regions including the ones in geometrically non-trivial shape (*i.e.*, other than just a circular region) are considered as well, the optimal join region 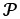 is computed based on the sizes of *R*, *S*, and *R* ⨝ *S* assuming that the network is *dense* enough for a node to be found at any point in the network. 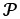 is formed using three points *C_r_*, *C_s_*, and *C_q_* which are related respectively to region ***R***, region ***S***, and the query sink *Q*. They lie within the triangle Δ***RS****Q*. Figure 4(b,c) show two possible shapes of 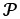. In Figure 4(b), 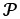 is formed of two curved paths <*C_r_*, *C_q_*> and <*C_s_*, *C_q_*>, a line segment $\bar{C_{q}Q}$, and a circular region *P_O_* of appropriate radius around *Q*. In Figure 4(c), 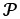 is formed of two curved paths <*C_r_*, *C_q_*> and <*C_s_*, *C_q_*>, and only a *part* of the line segment $\bar{C_{q}Q}$ (without the circular region around *Q* of Figure 4(b)).

In the case of not sufficiently dense networks, the join region *P* is defined based on the paths traversed by location routing protocols GPSR and TBF. TBF (trajectory-based forwarding) protocol [32] could be used in particular to traverse the curved paths <*C_r_*, *C_q_*> and <*C_s_*, *C_q_*> of the optimal join region 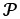. For reasonably dense sensor networks, the actual join region *P* yielded would be close to the originally intended optimal join region 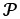.

In the second query model, to perform a sliding window join, both w̃*_R_* and w̃*_S_* are maintained in the join region *P*. A difference from the static join case is that not only *R* but *S* is shipped and distributed in *P* in the unit of a sliding window. w̃*_R_* (w̃*_S_*) is supposed to be partitioned among the nodes of *P* such that each node of *P* stores a partition of w̃*_R_* as well as a partition of w̃*_S_*. Each tuple *t* of *R (S)* is sent to *P* when it is produced. *t* is broadcast to every node of *P* such that it is joined with w̃*_S_* (w̃*_R_*). In this process, *t* is stored at the first encountered node of *P* with available memory. The join result tuples are sent to the query sink. The stream version of *Distributed-Broadcast Join* is a *symmetric* nested-loop join. The set of newly produced tuples of *R* (*S*) is the outer relation while w̃*_S_* (w̃*_R_*) plays the role of the inner relation. In computing the location of the optimal join region 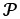, the size of 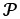 needs to be set so that both w̃*_R_* and w̃*_S_* could be stored in 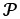.

*Distribute-Broadcast Join* can be effective for a long-running continuous join. Reliable statistics required in computing the optimal join region such as the sizes of *R*, *S*, and *R* ⨝ *S* can be collected when the query is long-running. It is especially so when data rates of *R* and *S* and join selectivity vary during the lifetime of the query. *Distributed-Broadcast Join* can adapt to node failures in traversing the join region because GPSR and TBF protocols can adapt to node failures, guaranteeing data delivery as long as a route exists.

#### Mediated Join

In [4], Coman *et al*. proposed *Mediated Join* to answer an inter-region join *R* ⨝ *S* to be executed as a one-shot query submitted at the query sink *Q*. It collects *R* and *S* at a join region *P*, performs the join, and sends the result to *Q. P* consists of one or more nodes, and its location is other than that of region ***R*** or ***S***. A detailed description of a distributed join algorithm in WSNs with which *Mediated Join* can be implemented is well presented in [3]. *R* ⨝ *S* is implemented in nested-loop join. First, *R* is distributed in *P*, and then *S* is broadcast to *P* for join. *R* and *S* respectively play the role of the inner and the outer relation.

In [3], Coman and Nascimento investigated a problem of optimal selection of *R* or *S* as the *outer* relation (with the remaining one being the inner relation). The choice would make a difference in communication cost of executing the same join. Depending on the choice, the size of the join region would be different. The size of the join region affects the cost for distributing the inner relation as well as the cost for broadcasting the outer relation. In [3], the cost formula that estimates the total communication cost *E* in processing *R* ⨝ *S* given the sizes of *R_i_* (inner relation) and *R_o_* (outer relation) is derived. Using this formula, it was shown that the total cost would vary considerably depending on which of *R* or *S* is selected as the outer relation. With *E*(*R_i_* = *R*) denoting the total cost where *R* is the inner relation, and ǁ*R*ǁ the size of *R*, it was shown that the ratio *E*(*R_i_* = *S*)/*E*(*R_i_* = *R*) non-monotonically varies from 0.6 to around 1.6 when the ratio ǁ*S*ǁ/ǁ*R*ǁ falls in the range [1,7]. If *E*(*R_i_* = *S*)/*E*(*R_i_* = *R*) is greater than 1, *S* is chosen as the outer relation. Otherwise, *R* is chosen as the outer relation. Once the routing tree of region ***R*** (***S***) is constructed, the size of *R* (*S*) can be collected at the root. Therefore, it is possible to pick a better nested-loop join strategy at the join region where the join is to be conducted.

#### Distributed Index-Join

In [8], Pandit and Gupta proposed an index join algorithm using a *distributed B^+^ tree* implemented in WSNs to answer a continuous join query. It executes an inter-region theta-join *R* ⨝*_Pr_ S*, where *P_r_* is a *range* predicate of the form, |*R.A.−S.B.*| *θ* for a value *v*. The join is modeled as a sliding window join. w̃*_R_* and w̃*_S_* are indexed on their joining attributes using a distributed B^+^ tree. It is created across the sensor nodes of the network as follows: First, a sensor node *N_R_* to store the root node of the B^+^ tree is chosen, and the degree (*i.e.*, branch factor) *d* of the B^+^ tree is determined. The proper value of *d* is set depending on the amount of memory available in a sensor node. Once *N_R_* and *d* are determined, a set *S_N_* of the sensor nodes including *N_R_* to store the B^+^ tree nodes are chosen. Each node in *S_N_* is assigned a value *range* of the joining attribute. This assignment process is conducted from *N_R_* to its descendants level by level, and the leaf nodes of the B^+^ tree are supposed to store the tuples of w̃*_R_* and w̃*_S_*. In a conventional B^+^ tree, a pointer to a child node or a sibling link of a leaf node is the memory address of the target node. In the proposed B^+^ tree in WSNs, those pointers are represented by the geographic coordinates of the *sensor node* that stores the target B^+^ tree node. Thus, access to the target node is carried out by a location routing protocol like GPSR.

Join processing proceeds as follows: When a tuple *t* of *R* (*S*) is produced, the tuples of w̃*_S_* (w̃*_R_*) that satisfy *P_r_* with *t* are searched from the B^+^ tree index, and *t* is stored at the leaf node of the B^+^ tree where it belongs. *Distributed Index-Join* is a symmetric *indexed* nested-loop join, where the set of newly produced tuples of *R* (*S*) is the outer relation while w̃*_S_* (w̃*_R_*) plays the role of the indexed inner relation. Since the join predicate is a range one, the leaf node of the B^+^ tree where the smallest value satisfying the predicate resides is searched, and then, through the sibling links, the subsequent leaf nodes are traversed until the largest value satisfying the predicate is reached.

In *Distributed Index-Join*, node failures are handled by data replication. A B^+^ tree index node *N_B_* stored in a sensor node is replicated at nearby sensor nodes. In the parent node of *N_B_*, the geographic coordinates referring to *N_B_* is now supposed to refer to a set of duplicated children. When the B^+^ tree is searched and *N_B_* is to be accessed, one of *N_B_*'s duplicates can be chosen.

*Distributed Index-Join* can be effective for a long-running continuous join. The cost of building and maintaining (e.g., evicting the old tuples outside the indexed sliding window) the distributed B^+^ tree index could be paid off for a long running join.

#### Distributed Hash-Join

In [8], Pandit and Gupta proposed *Distributed Hash-Join* to answer a continuous join query. It executes an inter-region theta-join *R* ⨝*_Pr_ S*, where *P_r_* is a *range* predicate of the form, ǀ*R.A*. − *S.B*ǀ *θ* for a value *v. Distributed Hash-Join* adapts the hash join to WSNs. The join is modeled as a sliding window join. It partitions w̃*_R_* and w̃*_S_* using the same hash function on the joining attributes. The join works as follows: In case *θ* is equality (*i.e.*, an equi-join), it hashes a newly produced tuple *t* of *R* (*S*) to geographic coordinates *c. t* is routed to the sensor node *N_c_* that is nearest to *c* using a location routing protocol like GPSR. Join tuple matching for *t* is done at *N_c_* because only the partition of w̃*_S_* (w̃*_R_*) which was hashed to *c* and placed at *N_c_* stores the tuples joinable with *t*. If the memory overflow occurs at *N_c_* when *t* is to be placed there, *t* is stored at the neighbor nodes of *N_c_*. *N_c_* keeps a record on its overflow area, maintaining it up-to-date afterwards.

In case *θ* is inequality (*i.e.*, a range query), a *locality preserving* hash function is employed. It hashes the tuples whose joining attribute values are in close proximity with each other to the sensor nodes located near to each other. Join processing for the range predicate is performed across these nodes.

In *Distributed Hash-Join*, node failures are handled by data replication. Suppose a node *N* stores the partitions of sliding windows w̃*_R_* and w̃*_S_*. The tuples in those partitions are replicated at nearby nodes. Unless not all of them including *N* fail, at least one active node that is closest to the hashed geographic coordinates is guaranteed to have the tuples stored in *N*.

#### Pair-wise Join

In [22,23], Mihaylov *et al*. considered a long-running continuous join whose expression could be as general as the following:

(1) $$(\sigma_{\phi_{R}\Lambda \Psi_{R}}R){⨝}_{\phi_{RS}\Lambda \psi_{RS}}(\sigma_{\phi_{S}\Lambda \psi_{S}}S).$$

∅_R_, *Ψ*_R_, ∅_S_, and *Ψ*_R_ are *selection* predicates, and ∅*_RS_* and *Ψ_RS_* are *join* predicates. The join is modeled as a sliding window join. Selection predicate ∅ is only on the auxiliary attributes which are *static* (e.g., node ID, the coordinates of an immobile node, *etc.*). Selection predicate *Ψ* is related with at least one sensor attribute. Similarly, the join predicates ∅ and *Ψ* are on the static and on the sensor attributes, respectively. For example, a join which is a modified version of an example query in [22], can be given as follows:

(2) $$\left(\sigma_{ID<25\;\Lambda \;A>200\;}R){⨝}_{R.x=S.y+5\;\Lambda \;R.A=S.B}(\sigma_{ID>50\;\Lambda \;B>200\;}S\right)$$

where *ID* is the node ID, *x* and *y* represent the coordinates of a node, *A* and *B* are respectively the sensor attributes of *R* and *S*.

**Figure 5.**
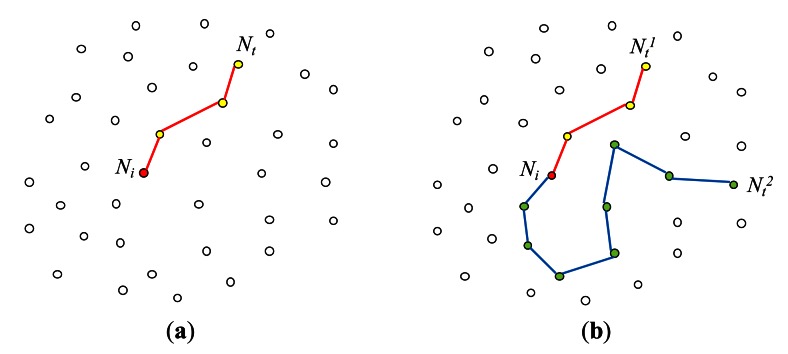
(**a**) An initiator node *N_i_* and its target node *N_t_*. A join between *N_i_* and *N_t_* is called a *pair-wise join*. (**b**) Two target nodes *N_t_*^1^ and *N_t_*^2^ for one initiator node *N_i_*. Each of the (*N_i_*, *N_t_*^1^) and (*N_i_*, *N_t_*^2^) pairs represents a pair-wise join.

Because of the spatial join predicate on the static attributes *x* and *y* (*i.e.*,*R.x* = *S.y +*5), a node that has received this query cannot determine if it is to participate in the query without communicating with other nodes. In [23], the problem of optimal join initiation for a query *q* with a spatial join predicate ∅*_RS_* was investigated. Each node *N_i_* that has received *q* searches the network through *multicasting* for its join counterpart node *N_t_* which satisfies ∅*_RS_* with *N_i_*. *N_i_* is called an *initiator node*, and *N_t_* is called a *target node* (Figure 5(a)). Note that for each initiator node, there could be multiple target nodes (Figure 5(b)). While *N_i_* searches for its target nodes, the vector information on the current path traversed so far is maintained so that backtracking to *N_i_* is possible. Once a target node *N_t_* is found, *N_i_* and *N_t_* communicate with each other, setting up the optimal in-network strategy of joining the tuples in *N_i_* with those in *N_t_*. In [22], this join between two nodes *N_i_* and *N_t_* is called a *pair-wise join* (or *1:1 join*). Since there could be multiple target nodes for each initiator node, there could be multiple pair-wise joins that contribute to the result of *q*. In [23], join initiation is carried out for each pair-wise join individually. As for multiple join pair optimization, it is carried out after individual join initiations are completed. It is described shortly.

**Figure 6.**
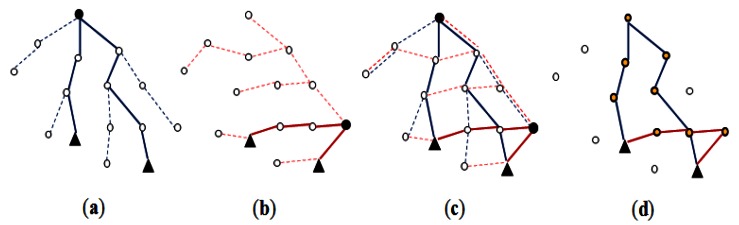
(**a**) A routing tree where a dark circle denotes the root, and the *join path* between two triangular nodes; (**b**) Alternative routing tree for the same set of nodes and a different join path; (**c**) The two routing trees are in use together, and there are two join paths searched; (**d**) Every node on the two join paths are candidate *join nodes*.

In [23], *multiple routing trees* are considered to provide several alternative paths between an initiator node and a target node. Using only one routing tree, the number of hops between two nodes physically near each other may be large. This problem can be handled by employing multiple trees. They can also reduce hot spots and congestion. Since multiple routing trees are employed, a target node may hear from the initiator node through multiple paths. The two nodes choose one of these paths and one node on the chosen path as the *join node*. Figure 6 shows an example where two routing trees are employed. Here, a cost-based selection of the optimal join node is carried out, assuming that selectivity of each predicate *Ψ* is known and that the number of hops from a node to the base station is known [22].

**Figure 7.**
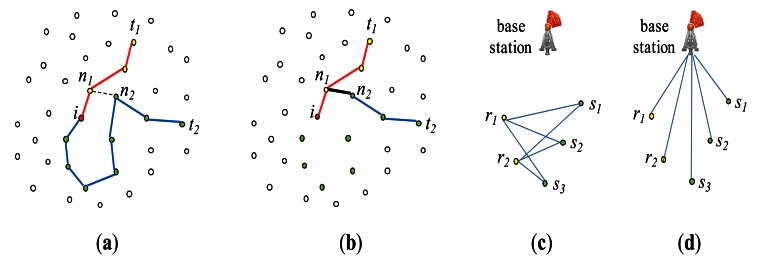
Multiple join pair optimization. (a) Before path collapse: there are two pair-wise joins (*i*, *t_1_*) and (*i*, *t_2_*). The two join paths are node-disjoint except *i*. There is a link between *n_1_* and *n_2_*; (b) After path collapse: the link between *n_1_*and *n_2_* is in use to make the join path of (*i*, *t_2_*) shorter; (c) Before group-based optimization for *R* ⨝ *S*, where ℝ = {*r_1_*, *r_2_*} and 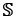 = {*s_1_*, *s_2_*, *s_3_*}; (d) After group-based optimization: *R* ⨝ *S* is executed as an external join.

In [22], the techniques of *multiple* join pair optimization were also proposed. One is network-level resource sharing. An example is a *path collapse* feature shown in Figure 7(a,b). For two pair-wise joins, if some part of their join paths could be shared to make one of the current join paths shorter, that path is updated. Another is a *group-based optimization* shown in Figure 7(c,d). For a join *R* ⨝ *S*, if a complete bipartite graph is formed between the two node sets ℝ and 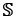 (*i.e.*, there are ǀℝ ǀ · ǀ 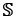
ǀ pair-wise joins running), the external join in which all the nodes in ℝ ∪ 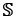 participate is considered as an alternative strategy. Cost analysis is carried out to check if the external join is cheaper than a group of ǀℝǀ ǀ 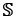
ǀ pair-wise joins.

In *Pair-Wise Join*, node failures are handled as follows: If a node on the join path fails after the path is established, the node of data source cannot reach the join node. Recovery is carried out by searching an alternative path. If such a recovery fails, a pair-wise join is switched to the external join. If the base station is unreachable from a node, it will wait for the routing trees to be rebuilt.

*Pair-wise Join* would be effective for the applications where each node that participates in the join query is expected to be paired with a small number of nodes due mainly to the spatial join predicates. It can be employed for a long-running continuous join. The statistics required in cost analysis for the optimal join initiation and in multiple join pair optimization are assumed to be collectable because the query is long-running. The optimal join initiation for a pair-wise join may be very costly, incurring high communication overhead. It could be amortized later if the query is a long-running one.

### 4.3. Techniques with Filtering of Non-Joinable Tuples

In this section, we present a survey of the join implementations named *Synopsis Join* [2], *Local Semijoin* [4], *INJECT* [21], *TPSJ* [6], *SENS-Join* [7], *CJF* [24], *PEJA* [5], and *SRJA* [12]. Their common feature is in-network filtering of non-joinable tuples.

#### Synopsis Join

In [2], Yu *et al*. proposed *Synopsis Join* to answer a one-shot join query. It executes an inter-region equi-join *R* ⨝*_A=B_ S*. A distributed variant of the semijoin is employed to fully reduce both *R* and *S*. Let *v-tuple* denote a tuple of *R* or *S* whose joining attribute value is *v*. Then, *v*-tuples of *R* are to be joined with *v*-tuples of *S*. The join proceeds as follows:

Each joining attribute value *v* of the *v*-tuples is geographically hashed to be sent to a sensor node *N_v_* located in a region *T* of the network, where the joinability of *v* is to be checked. The value *v* is sent as a part of the *synopsis* of a local relation at the sensor node where the corresponding *v*-tuple is stored. In [2], a *histogram* is used as an example of the synopsis, which stores <*v*, the count of *v*-tuples> pairs with other auxiliary information such as location information on the nodes storing the corresponding *v*-tuples. The location of region *T* is determined such that the transmission cost for sending the synopses to be imposed on region ***R*** would get roughly equal to that on region ***S***. Thus, *T* is located between ***R*** and ***S*** but closer to the bigger region of the two. If *v* turns out a joinable value (that is, *v* is in both *R*[*A*] and *S*[*B*]), *N_v_* notifies this to all the nodes of ***R*** and ***S*** storing the *v*-tuples. This notification is done with a location routing protocol like GPSR, for the location information of the *v*-tuples has been collected at *N_v_*. With this notification, *R* and *S* are fully reduced.

**Figure 8.**
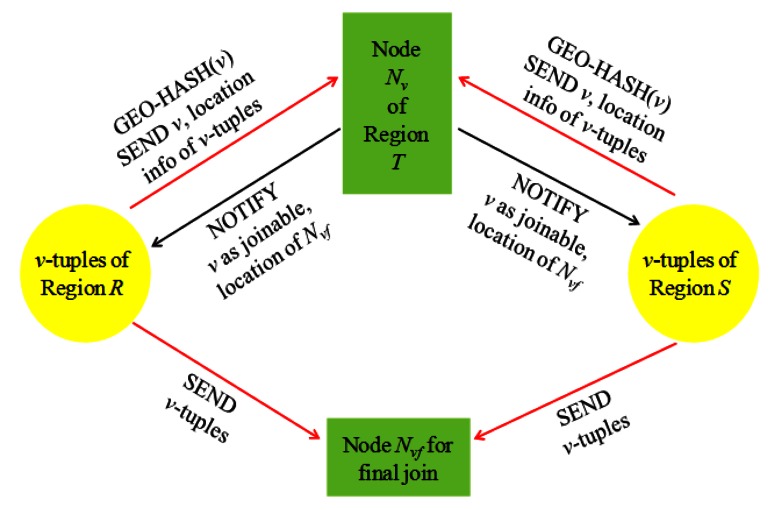
Non-joinable tuple filtering and final join for *R* ⨝ *S* using a distributed variant of a semijoin, where *v*-tuple denotes a tuple of *R* or *S* whose joining attribute value is *v*.

For each joinable value *v* in *R*[*A*] or in *S*[*B*], the final join among the corresponding *v*-tuples is conducted at a designated sensor node *N_vf_*, to which the *v*-tuples of regions ***R*** and ***S*** are sent. The final join result is sent to the query sink *Q*. The location of *N_vf_* is included in the aforementioned notification message sent by *N_v_*. Since *N_v_* has collected the location information of all the *v*-tuples of *R* and *S*, it can designate *N_vf_* such that the communication cost of the remaining steps can be minimized [2]. Figure 8 depicts the process of *Synopsis Join*, focusing on the aspect of non-joinable tuple filtering and the final join.

#### Local Semijoin

In [4], Coman *et al*. proposed the *Local Semijoin* to answer a one-shot join query. It executes an inter-region equi-join *R* ⨝*_A=B_ S*. A variant of semijoin is employed to fully reduce both R and S. In region R, the values of R.A and the IDs of their corresponding tuples are collected at the root of the routing tree. The root sends them to region S. At each node N in region S, semijoin R[A] ⋊*__A_θ_B__* S_N_ is conducted, where S_N_ is the subset of S stored at N. This fully reduces S. For the matched values of R[A], their corresponding tuple IDs are sent back to region R, reducing R fully as well. The two regions send those tuples that have survived to the query sink for the final join.

*Local Semijoin* is a direct application of the conventional semijoin to WSNs except for the *backward* reduction using the tuple IDs. In [4], however, it was shown that *Local Semijoin* always outperforms *Mediated Semijoin*, an alternative implementation which might seem a more general one. In *Mediated Semijoin*, a join region *T* possibly other than the two regions ***R*** and ***S*** is arranged to which *R*[*A*] and *S*[*B*] are sent along with their tuple IDs. The tuple IDs that have survived in *T* after *R*[*A*] ⨝_AθB_ *S*[*B*] are returned back to regions ***R*** and ***S***, fully reducing relations *R* and *S*. In [4], the cost formulas for *Local Semijoin* and *Mediated Semijoin* were developed assuming that the size of regions ***R*** and ***S*** is small. According to those formulas, the *optimal* location of *T* in *Mediated Semijoin* is shown to be that of either ***R*** or ***S***.

In *Local Semijoin*, data routing is carried out by GPSR protocol. It guarantees data delivery as long as a route exists and discovers new routes automatically when a node fails. In this sense, *Local Semijoin* can adapt to node failures as the underlying routing protocol does.

#### INJECT

Min *et al*. [21] proposed *INJECT* (In-Network Join strategy using Cost based optimization in Tree routing sensor networks) to answer a continuous join query. For a theta-join *R* ⨝ *S* which is to be executed periodically, *INJECT* performs cost-based optimization, selecting the optimal strategy for each execution. Each execution is against static relations *R* and *S* with the routing tree covering all the nodes of the network with the base station as the root. In *INJECT*, both semijoin results, *R* ⋊ *S* and *R* ⋉ *S*, are obtained inside the network. They are sent to the base station for the final join.

Beside the external join, *INJECT* considers various join strategies including *PartitionJoin*, *SynopsisJoin*, and *fullSynopsisJoin*. Before each execution of the query, *INJECT* estimates the communication cost of each strategy using a cost model. The optimal one is chosen and notified into the network. The cost formulas are derived using such statistics as the selectivity of a selection predicate and the semijoin selectivity. At first, these statistics are unknown. The external join is employed in the learning phases in which these statistics are collected.

**Figure 9.**
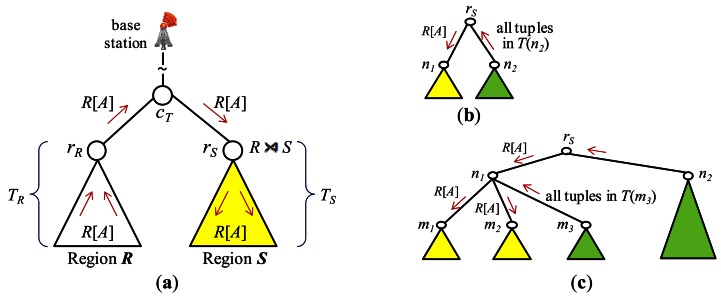
Semijoin *R* ⋊ *S* in *INJECT*. (**a**) Relevant portion of the routing tree for an inter-region join *R* ⨝ *S* and the shipping of *R*[A] for performing semijoin *R* ⋊ *S*; (**b**) Selective forwarding of *R*[A] in *PartitionJoin*; (**c**) Recursive application of selective forwarding of *R*[A] in *PartitionJoin*.

The basic idea of *PartitionJoin* is as follows: Let us consider an equi-join *R* ⨝*_A=A_ S*, for example. Regions ***R*** and ***S*** and their common path to the base station in the routing tree is shown in Figure 9(a). *T_R_* and *T_S_* respectively denote the subtree of region ***R*** and ***S***. A straightforward way to obtain the semijoin result *R*⋊ *S* at node *r_R_* (the root of *T_S_*) is to collect *R*[*A*] at node *r_R_* (the root of *T_R_*), forward it to *r_S_* via *c_T_*, and to filter out non-joinable tuples of *S* in *T_S_* by injecting *R*[*A*] into *T_S_* as shown in Figure 9(a). *PartitionJoin* optimizes this process as follows: *R*[*A*] is collected at node r_R_. It is forwarded to *r_S_* via *c_T_*. Non-joinable tuples of *S* in *T_S_* are filtered out by *selectively* forwarding *R*[*A*] into *T_S_*. In other words, *R*[*A*] is *not* forwarded to every node of *T_S_*. For example, suppose that there are two subtrees of *r_S_* (Figure 9(b)) and that *R*[*A*] is forwarded to the subtree whose root is *n_1_* (denoted as *T*(*n_1_*)), whereas it is not to *T*(*n_2_*). Then, all the tuples of *S* in *T*(*n_2_*) are supposed to be sent to *r_S_* where they are semijoined with *R*[*A*]. The reason why *R*[*A*] is not forwarded to *T*(*n_2_*) is that the cost of forwarding *R*[*A*] to *T*(*n_2_*) would be higher than the benefit of reducing the set of *S* tuples in *T*(*n_2_*). For the subtree to which *R*[A] is forwarded (e.g., *T*(*n_1_*)), such *selective forwarding* is *recursively* applied. Figure 9(c) shows the three subtrees of *T*(*n_1_*). *R*[A] is forwarded to *T*(*m_1_*) and *T*(*m_2_*), but *not* to *T*(*m_3_*). In [21], Min *et al*. proposed a dynamic programming algorithm that obtains the optimal selective forwarding of *R*[A] to *T_S_*.

*SynopsisJoin* and *fullSynopsisJoin* are the same as *PartitionJoin* except that a Bloom filter [31] of the joining attribute is forwarded as a synopsis instead of the values of the joining attribute. Since a Bloom filter is much smaller, the cost of the semijoin is decreased while the benefit could be increased. *SynopsisJoin* and *fullSynopsisJoin* are regarded as efficient adaptations of the conventional Bloomjoin [33] or hash-semijoin [34,35] to WSNs.

The in-network strategies of *INJECT* can be employed only in the case that each of the nodes *r_R_*, *c_T_*, *r_S_* in Figure 9(a) is equipped with the memory whose capacity is sufficient enough to buffer the data it is supposed to receive and hold. For example, with *SynopsisJoin*, *r_S_* should be able to store *R*[A] as well as *R* ⋊ *S*.

#### Two-Phase Self-Join (TPSJ)

In [6], Yang *et al*. proposed *TPSJ* to answer a continuous join query. It executes a theta-join query *q* of the form:

(3) $$(\sigma_{P}\;R){⨝}_{A\theta B\;\Lambda \;T(h)}R$$

where *P* is a selection predicate on the sensor relation, and *T(h)* is a temporal predicate to specify a time-based sliding window [19] where *h* is the window size. The semantics of *q* is defined as follows: Let *R*_1_ = σ_P_*R. R*_1_ is a set of tuples produced at the same time instance, say, at *t* throughout the sensor network. Then,

(4) $$q=R_{1}{⨝}_{A\theta B\;\Lambda \;T_{2}(t,h)}R,$$

where the temporal predicate *T*_2_(*t,h*) restricts *R* to those tuples produced in the time interval [*t*, *t*+*h*]. The join predicate *AθB* is checked between the tuples of *R*_1_ and those of *R* satisfying *T*_2_(*t,h*).

The join processing proceeds in two phases after *q* is rewritten at the base station by *decomposing q* into two subqueries *q*_1_ and *q*_2_, where *q*_1_ = σ_P_*R* and *q*_2_ = *R*_1_⨝*_AθB_*_Λ T2(t,h)_*R*:

- **Phase 1.** *q*_1_ is injected into the network, *R*_1_ is obtained and sent to the base station. Note that *R*_1_ received at the base station satisfies selection predicate *P* but has *not* been checked for join predicate *AθB*.
- **Phase 2.** The base station broadcasts *q*_2_ and *R*_1_ to the network. At each node *N*, the semijoin *R*_1_ ⋊ *_AθB_R^N^* is carried out, where *R^N^* is the subset of *R* produced at *N* in the time interval [*t*, *t*+*h*]. This process fully reduces the sliding window of *R* (to be exact, σ_*T*_2_(*t,h*)_ *R*) to *R*_2_. *R*_2_ is sent to the base station where *R*_1_ ⨝ *_AθB_ R_2_* is conducted to obtain the final join result.

*TPSJ* is devised to detect events in monitoring applications. With a selection predicate, the candidate sensor readings (*i.e.*, *R*_1_) that represent the event of interest are collected first. Then, *R*_1_ triggers the checking of the subsequent readings to see if they are correlated with *R*_1_. *TPSJ* would be effective when the selection predicate *P* is highly selective, because all the tuples of *R*_1_ are sent to the base station in the first phase.

In *TPSJ*, node failures are handled by restructuring the routing tree. When a node fails to hear from its parent for some period of time, it chooses a new parent. For proper restructuring, each node maintains information on its neighbor nodes such as the number of messages that it has received from a neighbor and the number of hops that a neighbor is away from the root. Each node also periodically broadcasts a message to its neighbors to inform them that it is active.

#### SENS-Join

In [7], Stern *et al*. proposed *SENS-Join* to answer a one-shot join query *q* = *R* ⨝ *S* whose join condition could be any form of *arbitrary predicates* on *n* joining attributes. In [7], the sensor relations *R* and *S* are modeled as storing one tuple per node (*i.e.*, the most recent measurement). The result of *q* is obtained in three steps after all the sensor nodes relevant to *q* form a routing tree with the base station as the root:

- **Step 1.** All the joining attribute values are collected at the base station, where the join condition is checked to filter out non-joinable values.
- **Step 2.** The *join filter* consisting of those values that have survived is constructed and broadcast to the network, where each node filters out non-joinable tuples by looking up the join filter.
- **Step 3.** The remaining tuples considered joinable are sent to the base station where the final join is conducted.

**Figure 10.**
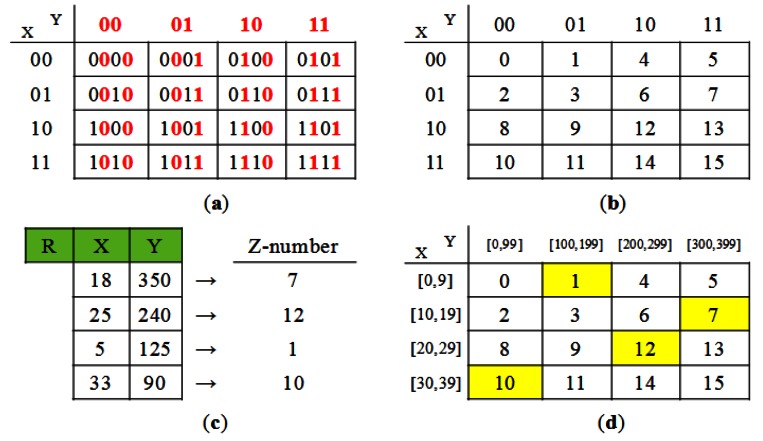
Z-ordering and its application for encoding joining attribute values. (**a**) Z-numbers in bits. The bit string in a cell is obtained through bit interleaving: one bit from *x*-dimension, and the next bit from *y*-dimension, and so on; (**b**) Z-numbers in decimal; (**c**) Relation *R* with 2 joining attributes *X* and *Y*, where value range of *X* is [0, 39] and that of *Y* is [0, 399]. Their subranges are specified in (d); (**d**) Mapping of the tuples of *R* in (c) to the marked cells of the 2-dimensional space.

In the first two steps, the joining attribute values are sent or broadcast in an *encoded* and *indexed* form for considerable reduction of communication cost. The data structure employed for compact representation of the joining attribute values is a *quadtree*[36] constructed in connection with *Z-ordering*[37]. The encoding works based on the *quantization* of joining attribute values as follows: Suppose there are *n* joining attributes in *q*. Each tuple can have up to *n* joining attribute values, which are mapped to a point in the *n*-dimensional space. For each dimension, its value range is divided into subranges of equal size. The number of subranges is a priori determined at the base station depending on the resolution of the dimension to be supported, and disseminated to the network independent of *q*. Then, a point in the *n*-dimensional space belongs to an *n*-dimensional cell. Each cell is numbered in Z-ordering (*i.e.*, assigned a *Z-number*), a technique which maps multi-dimensional data to one-dimensional data while preserving data locality. Figure 10 shows an example of Z-ordering for 2-dimensional cells and its application for encoding joining attribute values. Now a tuple *t* (to be exact, the *n* joining attribute values of *t*) is mapped to a Z-number. The Z-numbers of all the tuples are represented in a quadtree, which is known to be efficiently constructed in connection with Z-ordering.

Due to the *discrete* quantization of joining attribute values as described above, accuracy could be sacrificed in the first two steps while not undermining the correctness of the final join result in the third step. The degree of reduction of the join operand relation would be partial to full depending on the resolutions supported in encoding the join attribute values. It could be full reduction if the supported resolutions cover all the exact values of the joining attributes.

*SENS-Join* requires the routing tree to be stable throughout the execution of a query. In *SENS-Join*, link failure is handled. If a link fails during the execution of a query, the routing tree is repaired to be restructured. Then, the query is re-executed.

#### Continuous Join Filtering (CJF)

In [24], Stern *et al*. proposed *CJF* to answer a continuous join query. It periodically executes a theta-join query *q* = *R* ⨝ *S*. Each execution is against static relations *R* and *S*. In [24], the sensor relations *R* and *S* are modeled as storing one tuple per node (*i.e.*, the most recent measurement). In each execution, the final join result is computed at the base station. The *ideal* scenario is that each node of *R* and *S* sends its tuple *t* to the base station if *t* is joinable and does not send *t* if *t* is not. Then, the base station computes the join result with the received tuples. The goal of *CJF* is to get close to such an ideal scenario as follows:

At each node, a *join filter* (or simply a filter) on the joining attribute, say, *A*, is installed. The filter is represented as an *interval* [*v_1_*, *v_2_*] of the values of *A*. For ease of explanation, let us consider an equi-join query *q* on one joining attribute *A*: q = *R* ⨝*_A=A_ S* (*CJF* can process a theta-join on *k* joining attributes. The filter in each node consists of *k* intervals, one for each joining attribute. In each execution of *q*, each node sends its tuple *t* to the base station if *t.A* is outside (the interval of) the filter. Otherwise, it filters out (does *not* send) *t*. The base station computes the final join result with the received tuples. For correctness, however, the base station also checks if the value of *A* of any received tuple belongs to the interval of the filter in any node (say, *N*) that has not sent its tuple (say, *u*). This is called a *collision*, and it indicates the join possibility. If a collision occurs, the base station retrieves the missing tuple *u* by requesting *N* to send *u* to the base station. For this process, the base station needs to keep the filters of all the nodes. In fact, the filters of all the nodes are set by the base station before each execution of *q*. More description about this will be given shortly.

The interval of a filter [*v_1_*, *v_2_*] in node *N* is set using two values, *v* and *s: v_1_* = *v* – *s*/2, and *v_2_* = *v* + *s*/2. *v* is an estimate (*i.e.*, expected value) of *A* to be sampled in *N. s* is the *filter size* (*i.e.*, the size of the interval). A constraint in setting filters is that the filter intervals of two different nodes should not overlap. This is to ensure that the joinable tuples are not filtered out. For a pair of nodes *N_1_* and *N_2_* for which such a constraint cannot be enforced because the two tuples of *N_1_* and *N_2_* might be joined with each other, the filter is installed neither in *N_1_* nor in *N_2_*. That is, both filter sizes are set to 0. Then, *N_1_* and *N_2_* are supposed to send their tuples to the base station. That is, the filter size of a node whose tuple is joinable is set to 0 while that of a node whose tuple is not joinable is set as greater than 0.

The communication cost incurred in each execution of *q* depends on the filter sizes. Suppose tuple *t* produced in node *N* is not joinable. If the filter in *N* is too small, *t* might avoid the filter and be unnecessarily sent to the base station. If it is too large, the risk of collision at the base station gets higher. With this trade-off and the aforementioned constraint being considered, in [24], Stern *et al*. formulated the problem of determining the filter sizes for a given join query as a mathematical optimization problem, and proposed a solution.

In *CJF*, the base station computes the optimal filter sizes of all the nodes before each execution of the query. They are notified into the network for the filters to be maintained. In [24], it is assumed that the sensor readings would not drastically change between successive samplings. For example, the temperature in a region would not change much in a short period of time. Thus, the new filter sizes would not be much different from the previous ones. The filter updates are executed at some nodes only if communication cost for the update could be amortized in the next execution of the query.

As for the expected value *v* of *A* to be sampled at node *N*, the actual measurement would gradually diverge from *v* as time elapses. The update of *v* is triggered whenever a tuple is sent from *N* to the base station (*i.e.*, not filtered out). The new value is computed using a *linear regression model* with *k* recent values received at the base station. According to the experiments in [24], this model works well with *k* = 6. This implies that *CJF* can adapt to the trend of the sensor readings early enough.

*CJF* can be employed in the applications where an estimate of the sensor readings of each joining attribute could be provided, and also the sensor readings would not change much between successive samplings.

#### Progressive Energy-efficient Join Algorithm (PEJA)

In [5], Lai *et al*. proposed *PEJA* to answer a continuous join query. It executes an inter-region equi-join *R* ⨝*_A=B_ S*. The join processing proceeds as follows: In region ***R*** (***S***), a routing tree is constructed. In ***R*** (***S***), the value range of *A* (*B*) is divided into subranges. Each node of ***R*** (***S***) counts the number of value occurrences in *R.A* (*S.B*) for each subrange, making the *node histogram* which consists of a series of *(subrange, count)* pairs for that node. The node histograms are gathered at the root through the routing tree, and the root merges them to generate the *region histogram* which consists of a series of *(subrange, count)* pairs for that region. The two region histograms are sent to the node *c_T_*, which is located at the midpoint between the two roots of the routing trees. In *c_T_*, for each matching subrange pair, joinability for the subrange is checked. For example, if the count of a subrange [*v*_1_, *v*_2_] is not 0 in one region but that of the same subrange of the other region is 0, then there would be *no* join result for the subrange [*v*_1_, *v*_2_]. If both counts are non-zero, the join result is regarded to exist for [*v*_1_, *v*_2_] though this judgment would turn out to be a *false hit*. For the non-joinable subranges, the corresponding tuples of *R* and *S* are filtered out. Because of the false hits, such reductions of *R* and *S* are only *partial*. For each pair of joinable subranges, a strategy of in-network join processing is set up depending on the two counts. For example, if one count is high but the other one is very low, then the probability of the join result to be produced in that subrange could be low. If both counts are high, the probability could be high. Based on such a heuristic, a proper join strategy is selected and executed as follows.

The tuples of *R* that have survived are partitioned and replaced through geographic hashing. The region ***R*** is divided into *grids* where a grid is a rectangular geographic region of the network that comprises a set of sensor nodes. A subrange of *R.A* is mapped to the grids proportionally depending on the number of tuples in the subrange. For *I* which is a subrange of *A*, those tuples of *R* whose *R.A* belongs to *I* are sent to the grid *g* allocated for *I*. The same partitioning and replacement is conducted in region ***S*** for *B* as well, and the matching grid *g′*for *I* in ***S*** is called the *mirror grid* of *g*. Now the join tuple matching is carried out only between a grid and its mirror grid as in the conventional hash join. For each subrange *I*, the grid with less number of tuples send their tuples to its mirror grid where the final join is conducted. The matched tuples are sent to the base station for the final result. Each node of a grid *g* maintains a join filter *F_J_* that stores the joining attribute values in the mirror grid of *g. F_J_* is consulted in filtering out the non-joinable tuples in *g*. The joinable tuples of *g* matched through *F_J_* are sent to the base station, and *F_J_* of *g*'s mirror grid is updated accordingly.

*PEJA* can be regarded as an adaptation of the conventional *sort-merge* join, though *no* physical sorting of the tuples on the joining attribute is carried out in the network. The hash partitioning of the tuples is executed in association with partitioning of the joining attribute value range into subranges. This amounts to the *logical* (and *partial*) *ordering* of the tuples where the tuples in a subrange are not sorted.

#### SRJA (Synopsis Refinement iceberg-Join Algorithm)

**Figure 11.**
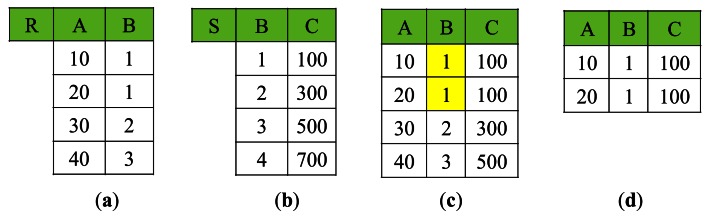
(**a**) Relation *R*; (**b**) Relation *S*; (**c**) Result of natural join *R* ⨝ _*B*=*B*_ *S*; (**d**) Result of iceberg join $R{⨝}_{B=B}^{\text{i}}S$ with iceberg threshold *α* = 2.

In [12], Lai et al. proposed *SRJA* to answer a one-shot join query. It executes an *iceberg join*[38] which is represented as an inter-region equi-join $R{⨝}_{A=B}^{\text{i}}S$. An iceberg join in WSNs is to find out the prominent patterns of correlation among the sensor readings. For a joining attribute value *v*, it contributes to the join result only if the number of joined tuples for *v* exceeds some given *iceberg thresholdα*. Figure 11 shows an example when *α* is 2.

In *SRJA*, each of regions ***R*** and ***S*** generates a *synopsis* of the joining attribute as follows. The value range of the joining attribute is divided into *k* subranges. For each subrange that covers, say, *n* values, the number of occurrences of each of those *n* values is counted. Among those *n* counts, the minimum and the maximum values are picked. Then, the synopsis of region ***R*** (***S***) is a series of *k* triples *(subrange, mincount, maxcount)*'s. The two region synopses are sent to the node *c_T_*, which is located at the midpoint between the two roots of the routing trees. In *c_T_*, for each matching subrange pair, joinability for the subrange is checked. For a subrange, if the product of the two corresponding maxcount's is less than *α*, then the subrange is flagged as *PRUNE*, because the tuples belong to that subrange do not contribute to the result. If the product of the two mincount's is greater than *α*, then the subrange is flagged as *JOIN*, because the tuples belong to that subrange might contribute to the result. In other cases, the subrange is flagged *DIVIDE*. The flagged synopsis of each region is sent back to the corresponding region where the tuples for the PRUNE-flagged subrange are filtered out. For the JOIN-flagged subrange, a variant of the semijoin is carried out between two regions to filter out non-joinable tuples, and the tuples that have survived are sent to the query sink for the final result. For the DIVIDE-flagged subrange, the corresponding value range is further divided, and the above process is repeated.

### 4.4. Summary and Assessment

Table 2 compares the techniques surveyed in Sections 4.2 and 4.3 where each row can be referred to as a summary of the corresponding technique. In Section 3.1, we have described the requirements and challenges in in-network join processing in WSNs. In the following, we assess the surveyed techniques in Table 2 in terms of those requirements and challenges. The major requirements include stream processing, distributed processing, protocol-oriented implementation, and fault-tolerance.

#### Stream processing

Among others, two important challenges are faced in meeting this requirement. One is to process a stream join with limited resources of the sensor nodes, and the other is to collect the statistics on the sensor readings for cost-based optimization. The state-of-the-art techniques have well met the first challenge: Most of the techniques can process stream joins [5,6,8,11,22,23]; The periodic executions of a join query can be regarded as a form of stream join [11,21,24]; Even the techniques that focus on a one-shot join over static join operand relations could be tailored to support a continuous join over streaming relations [2–4,7,12]. However, only a limited solution to the second challenge has been devised in the state-of-the-art. Most techniques do not employ a cost-based optimization based on the statistics. As for those employing cost formulas, the statistics are assumed to be collected through a learning phase in the processing of a long-running query [4,11,21,22].

#### Distributed processing

A difficult challenge is to generate an efficient distributed join strategy when each sensor node has limited knowledge of other nodes and the network connectivity. For inter-region joins, the join region is arranged around the center location of the triangle formed by the two regions and the query sink [3–5,8,11,21]. These techniques could be efficient when joining the sensor readings produced from two small regions of the network that are not overlapping. Other techniques employ the base station as a coordinator of join processing [6,7,24]. When all or a majority of the nodes participate in the query, these techniques could be more efficient than those of pure in-network processing because the base station is not resource-constrained. However, it might incur much communication when only small regions of the network distant from the base station participate in the query. The techniques that use geographic hashing distribute query operators over the network [8,2]. The communication costs of these techniques depend on the hash functions and the distribution of the values to be hashed. The join initiation in [22,23] is carried out in a distributed way using multiple routing trees. However, it is costly and could be amortized only for a long-running query.

#### Protocol-oriented implementation

A crucial challenge is to carefully design a protocol-oriented join implementation under the resource constraints of the sensor nodes. The state-of-the-art has well addressed this requirement. Examples of detailed description of protocol-oriented join implementations can be found in [2,3]. In [21], the lower bounds of memory space of a sensor node required in executing the semijoin strategies are shown. However, the full details of implementation are often missing in the state-of-the-art techniques. Some examples are as follows: In [2], the size of the region *T* where the filtering of non-joinable attribute values through geographic hashing is to be conducted is computed based on the available memory of a sensor node. However, a skewed value distribution that might cause memory overflow in a node in *T* is not considered. In [11], the available memory of a sensor node is considered in arranging the optimal join region, which can be obtained after the initial execution of the join at the query sink to which all the tuples are collected. If the query sink is a node inside the network, its memory space matters but such an issue is not addressed. In [8], the overflow at a node to which a tuple is routed through geographic hashing is addressed. But the details are not given.

#### Fault-tolerance

This requirement has received the least attention in the state-of-the-art. Though failure handling is addressed in several techniques, only a simple form of failure is treated in each technique. In [4,6,7,11,22], the successful transmission of data in spite of a node or link failure is considered. It is achieved through the handling of failures by their underlying routing protocols. In [8], the replication of sliding windows is considered. As for other techniques where failure handling is not addressed, the aforementioned failure handling could be incorporated to them. However, more critical forms of failures (e.g., a partial or total loss of the join nodes) have not been addressed in the state-of-the-art.

## 5. Open Issues

In the previous section, we have presented a survey of various techniques for in-network join processing in WSNs. Each technique could be enhanced to a more general solution with slight modifications. For example, the techniques proposed for one-shot join queries could handle a continuous query as well if the sliding windows were taken as join operand relations instead of the static snapshots of input relations. Other than such straightforward extensions, more issues need to be investigated for developing more advanced techniques. In this section, we discuss some of the open issues and future research directions.

For long-running continuous join queries, the statistics on the sensor readings required for cost-based optimization in the state-of-the-art techniques were assumed to be *sampled* from the results of prior executions rather than estimated. The effectiveness of such a practice could be limited if the query is not long-running. The initial execution in particular cannot be optimized. The techniques for estimating selectivity using sampling, histogram, and wavelet in the conventional database environment were investigated [39–42]. Those for estimating selectivity in data stream management systems were also investigated [43–47]. However, direct and in-network employment of these techniques in WSNs is infeasible due to communication overhead and resource constraints of the sensor nodes. Schemes of applying or adapting such estimation techniques to WSNs in connection with in-network join processing need to be investigated.

Cost-based optimization for one-shot queries or short-lived continuous ones might be intractable unless the statistics on the sensor readings are given a priori. A progressive and adaptive optimization could be considered for such queries. First, only an *initial* part of the strategy is determined. Then, depending on the intermediate result of join processing with the initial strategy and on the characteristics of the sensor readings thus far, the subsequent step is determined, and so on. In [34], Mullin showed that such an approach was feasible with hash-semijoins in distributed query processing. There are also a wide range of techniques for adaptive query processing, where the information that is obtained at query runtime is used to dynamically adapt the query plan [48]. Though these techniques were not targeted at WSNs, their adaptations to WSNs need to be investigated.

Join initiation for the queries with spatial join predicates (e.g., the predicate “*R.x* = *S.y* + 5” in query expression (2)) needs further investigation. Evaluation of such predicates could be carried out in the phase of join initiation or postponed to the phase of tuple matching. Evaluation of them as early as in the join initiation phase would reduce the number of sensor nodes participating in the join and restrict their possible pairings. It could make in-network join processing more efficient for long-running continuous queries. In general, this is a difficult problem. In [22,23], evaluation of spatial predicates in the join initiation phase is by an *exhaustive* search of node pairs that are to participate in the join using multiple routing trees. More efficient approaches to this aspect of join implementation need to be investigated.

The join queries considered in the literature is mostly a *2-way* join. In-network strategies suitable particularly for *n-way joins* were not addressed. Extension of the existing techniques to in-network strategies that can efficiently handle *n*-way joins is an open issue. In [49], Tran and Lee proposed a technique of employing semijoins for *n*-way window-based joins in a distributed data stream environment. Though it is not targeted at WSNs and the semijoin selectivities of the data streams are given as input parameters, the employment of semijoins has been shown to be efficient in an *n*-way join against distributed data streams. Adaptation of such an approach for a long-running continuous *n*-way join query in WSNs needs to be investigated.

Failure handling in the state-of-the-art techniques is not complete. This issue has not been dealt with thoroughly. Current solutions mostly handle a simple type of node or link failures only in some stages of in-network join processing. More critical failures such as the failure of the join nodes or the loss of intermediate join results were not addressed. A naïve approach to a complete solution might make join processing much more complicated and less energy-efficient. Adding fault tolerance to the existing techniques deserves more attention.

Another interesting open issue is to adapt the techniques developed for P2P databases. The current state-of-the-art surveyed in this paper is mostly under the influence of the techniques in the conventional centralized/distributed databases, focusing on the resource constraints of the sensor nodes in join processing and aiming at energy-efficient processing. Though the environments and technical goals of P2P databases are different from those of WSNs, they also provide distribution transparency, data independence through relational data modeling, and SQL-like relational query processing. Similar challenges are encountered in join processing on top of P2P networks such as the lack of statistics and peer/link failures. Relational query processors including various join implementations for such environments were proposed [50,51], and fault-tolerant query processing in P2P systems where the intermediate results in join processing are protected against failures was proposed [52]. Though they are not targeted at WSNs, their adaptations to WSNs deserve investigation.

Finally, join implementation and its evaluation in real-world WSNs are also important open issues. Most of the techniques surveyed in this paper have been evaluated in simulations. Since join selectivity is mostly assumed to be low, there is often no explicit statement about the required resources on individual nodes. In real-world settings, some techniques may need to be tailored because the assumptions made do not hold.

## 6. Conclusions

We have sketched the main ideas and features in the techniques of in-network join processing in WSNs proposed in the literature. We have described that join queries in WSNs are often characterized by spatio-temporal predicates. Spatially, an inter-region join is the most common type. Temporally, a long-running continuous join modeled as a time-based sliding window join is widely in use. Spatio-temporal characteristics have a direct influence on the design of in-network join implementation because different join types require different approaches to join implementation. Most of the join implementations are adaptations of the conventional join implementations in centralized and distributed databases. Some implementations filter non-joinable tuples out before the final join. Some conduct cost-based query optimizations.

The purpose of this survey has been to understand the existing join implementations, to classify join types, and to identify open issues for further development of advanced techniques. In-network processing of joins in WSNs will remain a significant area of active research because of its importance as well as its difficulty. It is hoped that this survey would contribute to a future breakthrough yet to come.

## Figures and Tables

**Table 1.** Notation.

**Symbol**	**Description**
*R*, *S*	Join operand relations for a join query *R* ⨝ *S*
ℝ ( 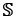 )	A set of sensor nodes which produce the sensor tuple stream of *R* (*S*). Each node in ℝ ( 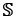 ) satisfies the predicates on geographic location of the nodes specified in the query.
***R*** (***S***)	A region of the sensor network that consists of the nodes in ℝ ( 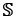 )
w*_R_*(w*_S_*)	A time-based window of *R* (*S*). It is either fixed or sliding.
w̿*_R_* (w̿*_S_*)	A time-based *fixed* window of *R* (*S*)
w̃*_R_* (w̃*_S_*)	A time-based *sliding* window of *R* (*S*)

**Table 2.** Comparison of various in-network join implementations.

**Techniques**	**Join Pred.**	**Query Type Supported^1^**	**Join Operand Relations^2^**	**Non-Joinable Tuple Filtering^3^**	**Involvement of Base Station^4^**	**Adapted Join Implementation**	**Routing Protocol**	**Optimization and Other Features**
*Distribute-Broadcast Join* [11]	theta	C, P, L	static, stream	NO	NO	nested-loop join	GPSR, TBF	cost-based selection of optimal join region
*Mediated Join* [3,4]	theta	O	static	NO	NO	nested-loop join	GPSR, tree routing	cost-based selection of inner/outer relation for nested-loop join
*Distributed Index-Join* [8]	range	C	stream	NO	NO	indexed nested-loop join	GPSR	dynamically creating and using a distributed B^+^ tree
*Distributed Hash-Join* [8]	equi, range	C	stream	NO	NO	hash join	GPSR	partitioning and joining tuples with geographic hashing
*Pair-wise Join* [22,23]	theta	C, L	stream	NO	NO	Orthogonal 5	multiple tree routing	cost-based join initiation for long-running join query, adaptivity (cost-based re-optimization)

*Synopsis Join* [2]	equi	O	static	full reduction of both relations	NO	semijoin, hash join	GPSR, tree routing	partitioning and filtering of the joining attribute values with geographic hashing
*Local Semijoin* [4]	theta	O	static	full reduction of both relations	NO	semijoin	GPSR	always outperforms *Mediated Semijoin* [4]
*INJECT* [21]	theta	C, P, L	static	partial or full reduction of both relations	NO	semijoin, Bloomjoin	tree routing	cost-based optimization
*Two-Phase Self Join* [6]	theta	C	stream	full reduction of one relation	YES	semijoin	tree routing	query decomposition assuming a highly selective selection predicate on one relation
*SENS-Join* [7]	general	O	static	partial to full reduction of both relations 6	YES	semijoin	tree routing	using quadtree as *n*-dimensional join filter based on Z-ordering
*CJF* (Continuous Join Filtering) [24]	theta	C, P	static	partial reduction of both relations	YES	ideal distributed join 7	tree routing	optimal join filter at each node
*PEJA* (Progressive Energy-efficient Join) [5]	equi	C	stream	partial reduction of both relations	NO	sort-merge join, hash join	GPSR, tree routing	logical sort through division of joining attribute value range, partitioning and filtering tuples with geographic hashing
*SRJA* (Synopsis Refinement iceberg Join) [12]	equi, iceberg	O	static	partial reduction of both relations	NO	sort-merge join, semijoin	GPSR, tree routing	logical sort through division of joining attribute value range

1 C = continuous, O = one-shot, P = periodic, L = long-running.

2 per window.

3 using a semijoin (or its variants) or a join filter.

4 other than in the final join.

5 The join method at the join node *N* is orthogonal to the proposed framework. Under the memory constraints at *N*, any centralized join implementation can be used.

6 where partial to full depending on the resolution of join filter.

7 a hypothetical distributed join method whereby only the joinable tuples of each site are sent to the final join site.
